# Age effects on transfer index performance and executive control in baboons (*Papio papio*)

**DOI:** 10.3389/fpsyg.2014.00188

**Published:** 2014-03-04

**Authors:** Elodie Bonté, Caralyn Kemp, Joël Fagot

**Affiliations:** ^1^Aix-Marseille University and CNRSMarseille, France; ^2^Laboratoire de Psychologie Cognitive, Aix-Marseille UniversityMarseille, France; ^3^Federation de Recherche 3C, Aix-Marseille UniversityMarseille, France; ^4^Brain and Language Research InstituteAix-en-Provence, France

**Keywords:** inhibition, cognitive flexibility, evolution, cognition, non-human primate

## Abstract

Reversal performance in the transfer index (TI) task is known to improve from prosimians to apes, suggesting that this task is a marker of cognitive evolution within the primate taxa (Rumbaugh, 1970). However, the cognitive processes recruited by this task remain unclear. In the present study, 19 socially-housed baboons *(Papio papio)* from 1.6 to 14.3 years of age were tested on a computerized version of the TI task, using an automated self-testing procedure. Age was a significant factor in the level of success, with the younger baboons outperforming the adults. The younger baboons learned the pre-reversal discrimination faster and improved their post-reversal performance more rapidly than adult baboons. As 17 of these baboons had already been tested in previous studies on inhibitory control and cognitive flexibility tasks, comparison across tasks provide indicators of the underlying cognitive processes. Age variations in performance were similar between the TI task and in an adaptation of the Wisconsin Card Sorting Task (WCST) measuring cognitive flexibility (Bonté et al., 2011). This contrasts previous results from a task requiring motor inhibitory control (Fagot et al., 2011). Therefore, these findings suggest that cognitive flexibility was a central component of the cognitive system that evolved within non-human primates. They also implicate a decline in executive control with age that begins during early adulthood in this baboon species.

## Introduction

The Transfer Index (TI) task (Rumbaugh, 1970, 1971; Rumbaugh and Pate, 1984) has been used extensively to compare cognitive performance, especially in non-human primates, in order to derive information on the evolution of intelligence. Subjects initially learn to discriminate between two visual stimuli, demonstrating attraction to the one associated with positive reinforcement (S+) while avoiding the negative stimulus (S−). Once discrimination is acquired up to a certain criterion level, a shift in reward contingencies occurs, with S+ becoming S− and vice versa. With this task, Rumbaugh (1970, 1971) first reported qualitative differences in learning processes between gorillas (*Gorilla g. gorilla*), gibbons (*Hylobates lar*) and talapoins (*Miopithecus talapoin)*: only the apes progressively learnt the concept of reversal with repeated exposure to new stimulus pairs (and their inversion), leading to accurate performance in the second, and all subsequent, post-reversal trials. This strategy was most evident when the apes were initially trained with a stringent learning criterion of 84% correct during pre-reversal trials, in comparison to the less stringent learning criterion of 67%. Talapoins, by contrast, used a more associative learning strategy to adapt to the new reward contingencies in post-reversal trials. This strategy was demonstrated by the reduced performance in the transfer trials in comparison to the apes, especially when the learning criterion achieved was high (84%).

Further assessments of the TI task in other non-human primate species have confirmed that a correlation exists between performance in the reversal trials of the TI task and phylogeny (Rumbaugh and Pate, 1984). Prosimians (Lemur: Rumbaugh and Arnold, 1971; Phaner Furcifer and Microcebus: Rumbaugh and Pate, 1984) and New World monkeys (Cebus apella: De Lillo and Visalberghi, 1994; Saimiri Sciureus: Rumbaugh and Pate, 1984) also showed lower post-reversal performance when trained in the TI task with the 84% learning criterion. Rhesus macaques (*Macaca mulatta*) obtained relatively mixed results in this task (Massel et al., 1981; Washburn et al., 1989). Apes more clearly applied rule learning, as demonstrated by an excellent performance that emerged as early as the second post-reversal trial (Rumbaugh et al., 1972; Rumbaugh and Gill, 1973; Gill and Rumbaugh, 1974; Rumbaugh and Pate, 1984). From these findings, Rumbaugh and his followers (Rumbaugh and Pate, 1984; Washburn and Rumbaugh, 1991; Gibson et al., 2001; Beran et al., 2010) proposed that the TI task may tap the key features leading to the emergence of intelligence and language in the primate order.

With the potential significance of the TI task to understand the evolution of cognition, it is important to determine which processes are involved in this task. Here we examined the hypothesis that the TI task might recruit two possible executive functions, cognitive flexibility and inhibitory control. Cognitive flexibility refers to the ability of subjects to attend to a shift in reward contingencies based on stimulus dimension, and to adapt their behavior in response to that shift. The Wisconsin Card Sorting Task (WCST) is considered to be the best test of cognitive flexibility (Berg, 1948; Royall et al., 2002; Stoet and Snyder, 2009). This test, which was successfully solved by non-human primates (Moore et al., 2003, 2006; Bonté et al., 2011) in an adapted version, requires participants to sort cards on the basis of a rule (e.g., select the red stimulus), and to subsequently switch to a new rule (e.g., now select the triangle) as the task contingencies periodically change.

In contrast, inhibitory control is defined as the ability to suppress potentially interfering thought processes or actions (Diamond, 2013). It is seen as a central component of human intelligence (Carlson et al., 1998; Dempster and Corkill, 1999). In laboratories, inhibitory control has been studied using a variety of different experimental designs, such as the stroop task (Stroop, 1992), requiring that the subject neglects (inhibits) the interference of one stimulus dimension to focus on another dimension, or the Stop signal task (Logan and Cowan, 1984; Logan, 1994), requiring the inhibition of an ongoing movement when a signal is produced. Several studies have shown that non-human primates are capable of solving adapted versions of the Stroop (Washburn, 1994; Lauwereyns et al., 2000; Beran et al., 2007) and Stop signal (Liu et al., 2009) tasks.

The TI task bears some resemblance with the WCST and, thus, presumably taps cognitive flexibility. Cognitive flexibility should be necessary, as these two tasks require that the subject abandon a previously rewarded response strategy to adopt a new one in the reversal trials. However, there are major differences between these two tasks. First, the WCST manipulates the stimulus dimensions (e.g., the color or shapes) while the TI task induces a shift in the reward contingencies of the entire set of stimuli. Second, the WCST involves more stimuli (traditionally 4; Berg, 1948) than the TI task (2; Rumbaugh and Pate, 1984). These two major differences suggest that the negative stimulus of the TI task exerts, presumably, a greater interference than the negative stimuli used in the WCST; this may potentially require stronger inhibitory control to counteract the interference induced by the distractors. Another important difference between the WCST and TI task is that the latter might require greater inhibitory motor control than the former due to a stronger associative strength related to the unique negative stimulus in the post-reversal trials.

The current study proposes that one way to disentangle the contribution of cognitive flexibility and inhibitory control in TI tasks is to investigate how these functions develop from childhood to adulthood in non-human primates. The few existing studies to have compared the efficiency of executive control by non-human primates over their lifespan suggest that cognitive flexibility and inhibitory control might not necessarily follow the same developmental trajectories in these species. On the one hand, Moore et al. (2006) has demonstrated that young macaques outperform macaques of middle age in a variation of the WCST. This effect was more recently replicated in a study of our own with baboons (Bonté et al., 2011), showing that 3–6 years old baboons outperformed their mid-adulthood counterparts. This effect was obtained in two versions of the WCST: one involving a shift in the stimulus dimensions rewarded, and a more complex task in which stimulus pairs were used with the subjects required to consider their abstract (same/different) relations.

On the other hand, the developmental profile of tasks requiring motor inhibition suggests a developmental profile different from the WCST. This effect is clearly demonstrated in Fagot et al. (2011; Experiment 2). In this study, baboons of different ages had to inhibit ongoing manual pointing toward a target stimulus as a consequence of a change in target location. Correct target responses varied between individuals from 5 to 67% correct, and were positively correlated with the age of the subjects. As the baboons tested in this study ranged in age from 2 to 14 years, this research did not demonstrate the performance decline that likely occurs in much older subjects. It nevertheless confirmed that inhibitory motor control tends to improve in efficiency from infancy to adulthood in baboons, in contrast to cognitive flexibility for which performance seems to decline during the same period (Moore et al., 2006; Bonté et al., 2011).

Within this theoretical context, the current study assessed reversal performance with a TI task in a troop of baboons in order to examine if performance in this task improves or declines from childhood to adulthood. It was reasoned that a negative relation between age and performance in the TI task would suggest that this task taps primarily cognitive flexibility as a core process, as already observed in an analog of the WCST in this species (Bonté et al., 2011). By contrast, a positive relation would suggest that motor inhibitory control is more central in this task, as found in Fagot et al. (2011). The results of this task can potentially inform us on how animals control their behaviors and how that control compares to that of humans. Further, it will provide new information on the development of executive functioning in non-human primates, and a reconsideration of the general significance of the TI task regarding the evolution of human intelligence.

## Methods

### Subjects and housing

Nineteen Guinea baboons (*Papio papio)* from a troop housed at the CNRS Primatology Center in Rousset-sur-Arc participated in this study. The group consisted of 5 males (mean age = 3.3 years ± 0.6) and 14 females (mean age = 8.9 years ± 4.7). Housing consisted of a 700 m^2^ enclosure with adjacent trailers containing the Automated Learning Devices for Monkeys (ALDM systems; see Fagot and Paleressompoulle, 2009; Fagot and Bonté, 2010). The baboons were fed once daily (monkey chows, vegetables and fruits) and water was provided *ad libitum*. The baboons had two biocompatible 1.2 × 0.2 cm Radio Frequency Identification (RFID) microchips in each forearm for automatic identification of the subjects by the testing systems.

### Stimuli

The stimuli used in this experiment consisted of 1000 (400 × 400 pixels) computer-generated geometrical shapes varying in color. These stimuli were randomly organized to create 500 pairs of stimuli.

### Apparatus

The baboons had permanent access to 10 ALDMs. Each ALDM is comprised of an open test booth (0.7 × 0.7 × 0.8 m), accessible from their enclosure, with a touchscreen that the monkeys could view through a small view port (7 × 7 cm) and touch by passing an arm through one of the two arm holes (8 × 5 cm). The RFID microchips implanted in each forearm allowed for automatic identification by the test system via antennas, fixed around each arm port. The experiment was controlled by a program written with Eprime language (v1.2, Psychology Software Tools, Pittsburgh, USA). This program allowed for an independent test of each baboon, based on their identity, regardless of the order in which each subject came to any of the test booths. Correct responses during the trials were food rewarded (dry wheat) using an automatic dispenser.

### Procedure

The experiment used a two-alternative forced-choice procedure inspired from the TI procedure of Rumbaugh (1970) and Rumbaugh and Pate (1984). Each started with the presentation of a stimulus pair, which appeared on the screen once the baboon's RFID microchip was read. The pair contained a positive stimulus (S+) and a negative stimulus (S−), randomly displayed on the right or left side of the touchscreen. Five seconds were allocated for a response, with a correct selection of the S+ stimulus resulting in the delivery of the food reward, while the selection of the S− stimulus, or no response, triggered a 3 s green screen time-out, with no food reward. An automated inter-trial interval of a minimum of 3 s was introduced between two consecutive trials; this time interval could be longer as it depended on the baboons' willingness to process the next trial.

The baboons were presented with one stimulus pair at a time, first in training trials and then immediately in reversal test trials when the required learning criterion had been met. In practice, the baboons were tested with 50 stimulus pairs with a fixed training criterion (of either 67 or 84% correct), after which the other test criteria was proposed for 50 new pairs. This procedure, in which the 67 and 84% criterion was alternated, was continued until all 500 pairs had been tested. Half of the baboons (randomly determined) were first tested with the 67% criterion and the other half with the 84% criterion. Computation of the learning criterion followed the standard procedure of Rumbaugh (1970); Rumbaugh and Pate (1984). Table 1 provides a summary of how these two criteria were computed during training. On specified trials (e.g., trial 11 for the 67% criterion), the program verified if the required number of correct trials had been performed (7 or 8 see Table 1 in this example) within that subset. The program continued the training trials if the number of correct trials performed by the subject was below the expected number. If the subject had achieved the performance requirement, the stimuli reversal trials were presented. However, in the situation that the baboon had exceeded the performance expectation (i.e., in our example, performed 10 or more correct responses), the program discontinued the test with the considered paired and presented a new pair of stimuli for the baboon to learn. When the learning criterion was reached, but not exceeded, testing was completed with a series of 11 reversal trials, in which the reward contingencies between S+ and S− were reversed. Many pairs were over-learned (*M* = 140 (*SD* = 68) and *M* = 107 (*SD* = 55) for the criterion 67 and 84%, respectively) and were consequently not followed by post-reversal testing. Reliable measures were obtained on a minimum of 40 pairs per baboon and learning criterion. To equate practice effects among the subjects and between the two learning criteria, the analyses focused on the first 40 pairs achieved by each baboon at each criterion level (80 pairs total per baboon).

**Table 1:** **Number of correct responses required (middle column) at each training trial (right column) to reach the 67 and 84% learning criterion**.

**Criterion**	**Number of correct responses**	**Trial number**
67%	7 or 8	11
	9	14
	10	16
	12	19
	14 in the last 21 trials	22–60
84%	9	11
	14	17
	17 or 18	21
	17 or 18 in the last 21	22–60

### Dependent variables and analyses

Learning abilities were measured using four dependent variables. The first of these was the number of trials required to reach the criterion for each pair, thus providing information on learning speed. The other three variables focused on the post-reversal trials. Firstly, post-reversal average scores were computed for each trial (trials 2–11) and learning criterion (67 and 84%). Note that the first reversal trial was not considered for these analyses, as the subject was not yet informed that the reinforcement contingencies were reversed. Secondly, we calculated the perseveration corresponding to the number of consecutive trials for each pair during which the baboon continued to touch the former S+ in the post-reversal phase, before its first attempt to touch the new S+. Finally, TI values were computed. Following the method developed by Rumbaugh and Pate (1984), TI was defined as the ratio between the percentage of correct responses achieved during post-reversal trials and the percentage correct of the pre-reversal trials (67 or 84%). All dependent variables were computed on the 40 pairs achieved at each criterion level.

## Results

### Pre-reversal trials

**Figure 1:**
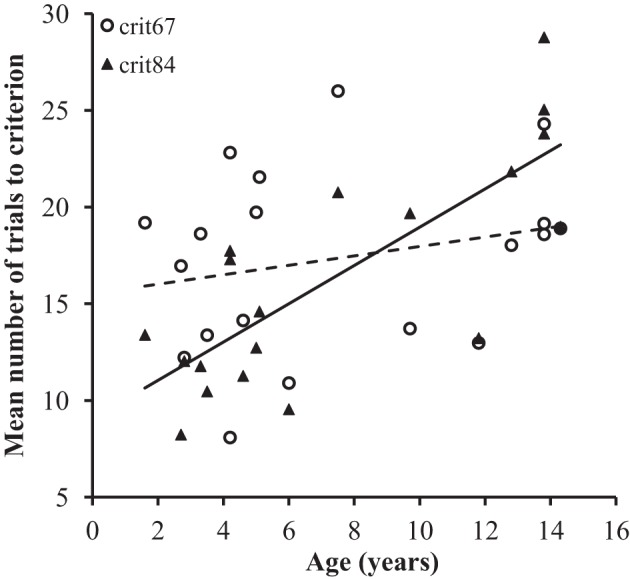
**Mean number of trials to achieve the criterion of 67 or 84% of correct responses as a function of the Age of the participants and Learning Criterion**. The dotted line corresponds to the criterion 67%.

The average number of pre-reversal trials required to reach criterion are presented in Table 2, for each subject. Analyses of these trials considered the 40 stimulus pairs acquired with each learning criterion (80 pairs total per participant). On average, the baboons required 17.3 trials to achieve the criterion of 67% and 16.4 trials for the criterion of 84%. Preliminary analyses found no significant effect of Sex on the dependent variables. An analysis of covariance (ANCOVA) was performed on the number of trials required to achieve the two learning criterion by Age. Learning Criterion (67 or 84%) and Set (4 sets of 10 pairs) were included as factors. Use of the Set as a factor was aimed at analyzing whether the learning speed increased with repeated testing. The main effect of Learning Criterion and Set were not significant *(p* > 0.05). In contrast, there was a significant effect of Age on the mean number of pre-reversal trials required to reach the learning criterion [*F*_(1, 17)_ = 10.6, *p* < 0.01]. However, this Age effect was accounted for by a significant two-way Age by Learning Criterion interaction of a higher level [*F*_(1, 17)_ = 11.2, *p* < 0.01]. As shown in Figure 1, the number of trials to criterion increased more drastically with age for the 84% than for the 67% criterion.

**Table 2:** **Participant name, sex, age, and performance data**.

**Baboons**	**Sex**	**Age**	**Learning speed 67%**	**Learning speed 84%**	**Ncorrect 67%**	**Ncorrect 84%**	**Perseveration**	**TI67%**	**TI 84%**
ANG	F	4.6	14.1	11.3	77.5	76.0	0.79	1.16	0.9
ARI	F	4.2	8.1	17.3	80.1	76.5	0.66	1.19	0.91
ART	M	4.2	22.8	17.7	82.3	78.3	0.78	1.23	0.93
ATM	F	11.8	13	13.2	75	74.8	0.78	1.12	0.89
BARB	M	3.5	13.4	10.5	76.2	79.6	100	1.14	0.95
BOB	M	3.3	18.6	11.8	86.5	79.3	0.78	1.29	0.94
BRI	F	13.8	24.3	28.8	40.8	37	1.35	0.61	0.44
CAU	M	2.7	17	8.2	83.3	79.5	0.55	1.24	0.95
CLO	M	2.8	12.2	12	85.3	82	0.83	1.27	0.98
DRE	F	1.6	19.2	13.4	80.5	76	0.65	1.2	0.9
KAL	F	14.3	18.9	19	61.5	66.5	1.66	0.92	0.79
LEA	F	13.8	18.6	23.8	55.5	44.3	1.48	0.83	0.53
MIC	F	13.8	19.1	25	41.3	45	1.58	0.62	0.54
MON	F	12.8	18	21.8	58.5	44.5	1.85	0.87	0.51
ROM	F	9.7	13.7	19.7	64.7	58.3	1.29	0.97	0.68
TAR	F	7.5	26	20.8	52.4	51.6	0.89	0.78	0.6
URA	F	6	10.9	9.6	81.3	76.5	1.03	1.21	0.9
VAN	F	5.1	21.6	14.6	78.5	81	0.64	1.17	0.96
VIO	F	5	19.7	12.7	73.7	73.1	0.96	1.1	0.85
	MEAN		17.3	16.4	70.2	67.3	10.2	1.05	0.8
	*SD*		4.7	5.8	14.5	15.2	3.9	0.22	0.18

*This table indicates, for each subject and training criterion, the mean number of trials required to reach each criterion (learning speed), the corresponding mean percentage of post-reversal correct responses, the mean number of perseveration trials, and the TI value*.

### Post-reversal scores

**Figure 2:**
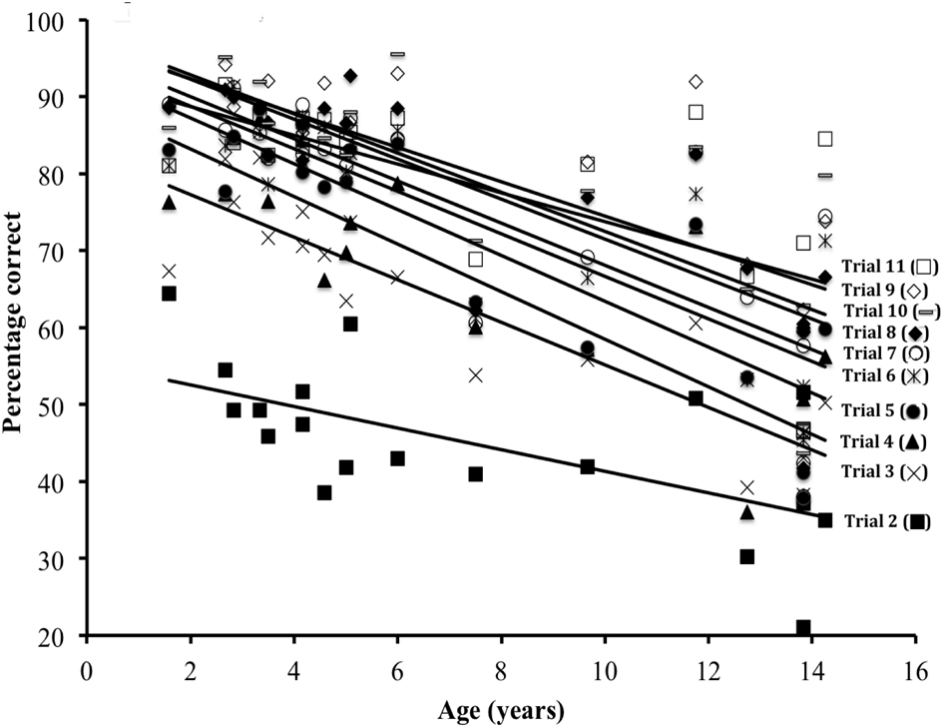
**Percentage correct as a function of the Age of participants and post-reversal trial number (from 2 to 11)**.

Arcsin transformed data were submitted to an ANCOVA using the variables of Trial (2nd to 11th after the reversal), Age, Learning Criterion and Set as factors. The Set factor was non-significant (*p* > 0.05), but the Trial [*F*_(9, 153)_ = 60.18, *p* < 0.001], Age [*F*_(1, 17)_ = 44.35, *p* < 0.001] and Learning Criterion [*F*_(1, 18)_ = 7.9, *p* < 0.05] had a significant effect on performance. The main effect of criterion revealed that the performance was higher on average for the 67% (*M* = 70.2, *SD* = 22.3) than 84% (*M* = 67.3, *SD* = 23.4) criterion. The main effects of Age and Trial interacted significantly [*F*_(9, 153)_ = 3.2, *p* < 0.01]. Figure 2 illustrates (for ease of viewing, we have used the non-transformed data), that the youngest subjects had a faster improvement of performance after trial 2, and reached higher scores with practice than their older counterparts.

### Perseveration trials

**Figure 3:**
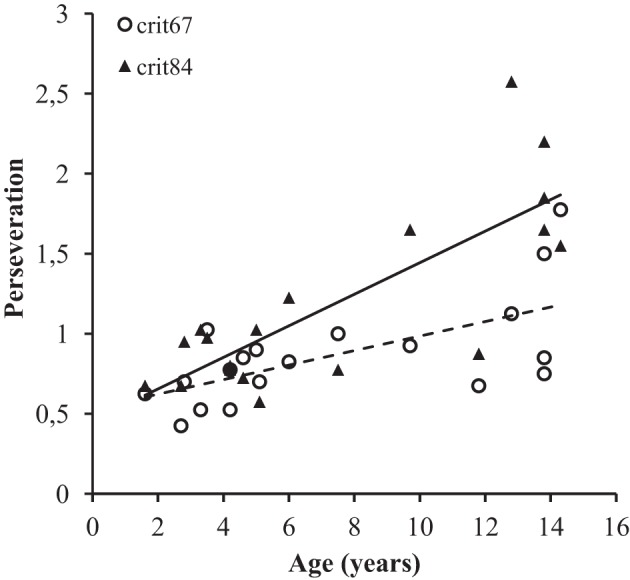
**Mean number of perseveration responses obtained during the post-reversal trials, as a function of the Age of participants and Learning Criterion**. The dotted line corresponds to the criterion 67%.

On average, the baboons made 1.03 perseveration trials (*SD* = 0.69) after the reversal of the stimulus reward contingencies, again discounting the first trial. An ANCOVA, using the same variables as above, revealed that the Set factor was not significant (*p* > 0.05). By contrast, the main effect of Age [*F*_(1, 17)_ = 42.02, *p* < 0.001] and Learning Criterion [*F*_(1, 17)_ = 9.28, *p* < 0.01] were significant. These latter factors, however, interacted significantly [*F*_(1, 17)_ = 5.05, *p* < 0.05], demonstrating that older individuals perseverated more than the younger baboons, especially when the 84% criterion was required (Figure 3).

### Transfer index value

**Figure 4:**
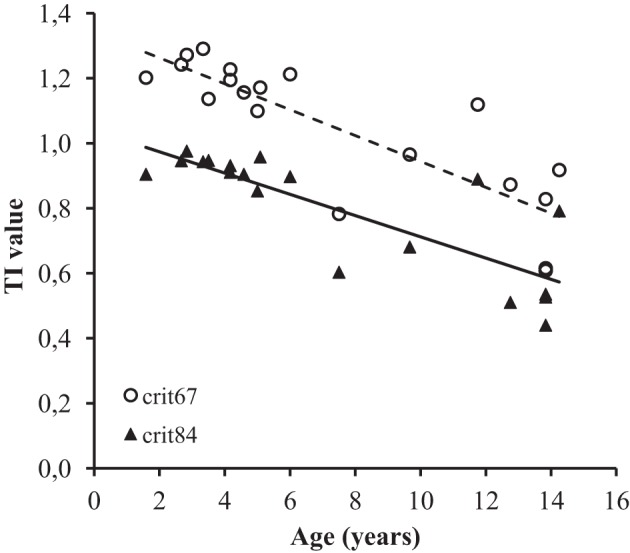
**TI values as a function of the Age of the participants and Learning Criterion**. The dotted line corresponds to the criterion 67%.

The final ANCOVA analysis considered the factor of Age, Learning Criterion and Set as independent variables and the TI values as the dependent variable. The effect of Learning Criterion was significant, *F*_(1, 17)_ = 246.59, *p* < 0.001. As shown in Figure 4, this main effect corresponded to higher TI value for the 67% criterion (*M* = 1.05, *SD* = 0.22) than for the 84% criterion (*M* = 0.80, *SD* = 0.18). The main effect of Age was also significant [*F*_(1, 17)_ = 40.1, *p* < 0.001]. On average, the TI values declined with the age of the participants (see Figure 4). Finally, none of the interactions were significant, although the Age by Learning Criterion interaction approached significance [*F*_(1, 17)_ = 3.95, *p* = 0.06].

## Discussion

Our data support the contention that the baboons exhibit a negative transfer in the TI task in relation to levels of pretest learning; therefore, their performance in the task was controlled by associative learning mechanisms more so than by forms of reasoning of a higher abstract level. Considering the group as a whole, the difference between the TI values in 67% and 84% condition was equal to −0.25. That value is roughly in the range of what has been previously reported for Old World monkey species (Washburn et al., 1989) and definitively lower than the positive values indicative of rule learning reported in apes (Rumbaugh and Pate, 1984). Therefore, this confirms the general idea that the TI task is an appropriate tool for revealing cognitive evolution within the primate taxa.

Detailing the relation between performance in the TI task and phylogeny is a first important step to understanding the evolution of cognitive systems. However, this information can only make sense if we can identify the cognitive mechanisms involved in the TI task. The main objective of this research was to describe in baboons the changes in TI performance during lifespan, in order to compare that developmental profile to already published data on age effects for tasks requiring cognitive flexibility or inhibitory control. Our findings show that younger individuals performed better in the present task than adults. Thus, the number of pre-reversal trials, as well as the number of perseveration trials, increased with age, more so for the more stringent 84% learning criterion. By contrast, the percentage correct in the post-reversal trials showed a negative relation with age, and was generally higher for the 67% learning criterion. It is important to note that these findings were obtained from subjects ranging from childhood to middle age, and therefore do not reflect the cognitive decline that likely occurs during old age.

Fagot et al. (2011, Experiment 2) had already tested 17 of the current baboons in a task requiring withholding and reorienting an ongoing pointing gesture in order to adapt to a change in stimulus location. Although 1 year elapsed between this first task and the current one, it is informative to assess if the number of perseveration trials of the present study correlated with the performance in the task of motor inhibition of Fagot et al. (2011): the correlation obtained on the 17 baboons who performed the two tasks was negative and significant (*N* = 17, *r* = −0.50, *p* < 0.05). This negative correlation suggests that these two tasks are not driven by the same cognitive process(es).

In another recent study from our group (Bonté et al., 2011), the same 17 baboons as above were tested in an adapted version of the WCST. Following the same reasoning as for the motor inhibition task, we computed the correlation between the numbers of perseveration trials in (Bonté et al., 2011) and those obtained in the current study. The results departed remarkably from the negative correlation of −0.50 reported for the inhibitory task: the correlation was positive and marginally significant (*N* = 17, *r* = 0.47, *p* = 0.06). Because the WCST is a marker of cognitive flexibility (Crone et al., 2004; Chevalier and Blaye, 2009), this finding is a first indication that TI task may be driven by cognitive flexibility skills.

Two independent studies further support the idea that cognitive flexibility has a reduced efficiency in adult monkeys in comparison to the younger individuals, with performance in both the WCST and the TI task being similarly influenced by age. First, using an adaptation of the WSCT task, Moore et al. (2006; but see Weed et al., 2008) reported that the number of perseveration trials was greater in rhesus monkeys during mid-adulthood than in younger individuals. Second, Kinoshita et al. (1997) reported a TI decline in Japanese macaques (*Macaca fuscata*), which started between the age of 3–5 years and continued in adults.

Neurobiological studies have shown that age-related decline in the neuroanatomy and neurochemistry of the brain are more evident in the frontal lobes—which coordinate executive functions—than in other cortical areas (Daigneault et al., 1992; West, 1996; Raz et al., 1997; Amieva et al., 2003). Evidence from neuropsychology suggests that older adults demonstrate a subclinical executive decline relative to younger individuals (Haug et al., 1983). Moreover, older people tend to perseverate more in tasks involving reversal learning (Daigneault et al., 1992; Dempster, 1992; Daigneault and Braun, 1993). A decline in executive performance would be expected in old monkeys, but our study intriguingly demonstrates that it occurs earlier during the baboon's life. Neuroanatomical structural changes with age were reported in the frontal cortex of rhesus macaques (Boese et al., 1982), but, to our knowledge, these modifications did not occur as early as observed here in baboons. Although different behavioral studies have also indicated an early decline of reversal performance in adult monkeys (Luebke et al., 2004), often because they perseverate more (Moore et al., 2003, 2006), conclusions regarding that early decline would remain premature at this point and warrant further investigation.

In more general terms, the current research has the main advantage of demonstrating that the TI task, a standard in the comparative cognition literature, probably measures the same kind of cognitive processes as the WCST, which is a standard test of executive function in humans. Use of the TI task has repeatedly demonstrated that the performance in this task varies within the primate phylogeny, with the apes out-performing the other non-human primates. Accumulative evidence suggests that intelligence correlates in humans with the efficiency of executive functioning (Engle et al., 1999; Brydges et al., 2012). These studies, in combination with the current one, suggest that the evolution of executive functioning, especially cognitive flexibility, was a critical factor that may explain inter-species variation in cognitive abilities. Future studies should attempt to further clarify this relation between the evolution of TI performance and cognitive flexibility.

## Authors' contributions

Elodie Bonté and Joël Fagot developed and ran the test design, Joël Fagot wrote the test program, Elodie Bonté conducted the statistical analysis, Elodie Bonté wrote the first draft; and all authors contributed equally to latter versions of this article.

### Conflict of interest statement

The authors declare that the research was conducted in the absence of any commercial or financial relationships that could be construed as a potential conflict of interest.
